# Inflammation, not Cholesterol, Is a Cause of Chronic Disease

**DOI:** 10.3390/nu10050604

**Published:** 2018-05-12

**Authors:** Alexandros Tsoupras, Ronan Lordan, Ioannis Zabetakis

**Affiliations:** Department of Biological Sciences, University of Limerick, V94 T9PX Limerick, Ireland; Alexandros.Tsoupras@ul.ie (A.T.) Ronan.Lordan@ul.ie (R.L.)

**Keywords:** cardiovascular disease, atherosclerosis, inflammation, platelet-activating factor, oxidised lipoproteins, cholesterol, chronic diseases

## Abstract

Since the Seven Countries Study, dietary cholesterol and the levels of serum cholesterol in relation to the development of chronic diseases have been somewhat demonised. However, the principles of the Mediterranean diet and relevant data linked to the examples of people living in the five blue zones demonstrate that the key to longevity and the prevention of chronic disease development is not the reduction of dietary or serum cholesterol but the control of systemic inflammation. In this review, we present all the relevant data that supports the view that it is inflammation induced by several factors, such as platelet-activating factor (PAF), that leads to the onset of cardiovascular diseases (CVD) rather than serum cholesterol. The key to reducing the incidence of CVD is to control the activities of PAF and other inflammatory mediators via diet, exercise, and healthy lifestyle choices. The relevant studies and data supporting these views are discussed in this review.

## 1. Introduction

### 1.1. Biological Significance of Cholesterol—Circulating Blood Cholesterol

Cholesterol, an unsaturated alcohol of the steroid family, is essential for the normal function of all animal cells. It is also a fundamental element for the normal structural makeup and the fluidity of all cell membranes. Cholesterol interacts with phospholipid bilayers in the cell membrane and increases membrane packing. Cholesterol also takes part in signal transduction, intracellular transport, nerve conduction, and signalling pathways through lipid rafts and caveolae. Cholesterol has various other biological functions, i.e., it is a precursor molecule for several biochemical pathways such as the synthesis of vitamin D, steroid hormones (e.g., cortisol, aldosterone, and adrenal androgens), and sex hormones (e.g., testosterone, oestrogens, and progesterone). Cholesterol is also a constituent of bile salts, which are crucial constituents of digestion, as they facilitate the absorption of lipids, fats, and fat-soluble vitamins A, D, E, and K [1].

Since cholesterol is mostly a lipophilic molecule, it does not dissolve well in blood. For this reason, it is packed into lipoproteins that are composed of a lipid core (which can contain cholesterol esters and triglycerides) and a hydrophilic outer membrane comprising phospholipids, apolipoprotein, and free cholesterol. This allows for the transport of the nonpolar lipid molecules such as cholesterol and triglycerides around the body through the blood to cells that require them. Plasma lipoproteins are separated into five major classes: chylomicrons, very-low-density lipoproteins (VLDL), intermediate-density lipoproteins (IDL), low-density lipoproteins (LDL), and high-density lipoproteins (HDL) [1,2].

Cholesterol can enter the blood through the digestion of dietary fat via chylomicrons. However, since cholesterol has an important role in cellular function, it can also be directly synthesised by each cell in the body. Notably, LDL particles are thought to act as a major transporter of cholesterol to the peripheral tissues, as at least two-thirds of circulating cholesterol resides in LDL. Conversely, HDL molecules are thought to do the opposite. They take excess cholesterol and return it to the liver for excretion [1,2].

Recent evidence suggests that dietary intake of cholesterol can influence plasma and serum levels, but not significantly. However, this is still subject to debate and further study [3]. Plasma cholesterol levels along with the levels of LDL cholesterol, HDL cholesterol, and serum triglycerides are currently used as biomarkers of the so-called standard ‘lipid profile’ for each individual. The standard lipid profile has been widely used as a traditional biomarker, not only for cardiovascular health but also for other lipid-related abnormalities and disorders [4].

### 1.2. Cholesterol Levels: Demonising a Risk Factor but Not the Causative Mechanisms of Chronic Diseases

Several modifiable and non-modifiable risk factors (genetic, environmental, nutrition, and lifestyle, etc.) are thought to influence the balance between health and disease by inducing mechanisms related to disease onset, development, and the manifestations of symptoms. The presence or coexistence of these risk factors seem to trigger underlying molecular and cellular mechanistic pathways that can lead to continuous chronic manifestations and the long-term loss of tissue homoeostasis and tissue dysfunction. These continuous chronic manifestations can develop over time before cellular disturbances manifest and cause tissue disorders, while, if not counterbalanced by our immune system and by specific preventive measures such as a healthy diet and lifestyle, the subsequent symptomatic disease finally appears, and medical treatment may be required to reduce the risk of mortality. Elucidating these molecular and cellular mechanistic pathways and acquiring the mechanistic evidence of the underlying multifactorial causes of a chronic disease can lead to suitable preventive targets against these diseases with fewer side effects, which is an ongoing difficult and demanding task. Such difficulties have misled the scientific and medical community to often and lightly extrapolate the easily acquired observed statistical and epidemiological correlations of traditional risk factors to several chronic diseases, towards matching these risk factors as the causative agents of these diseases.

According to the ‘cholesterol hypothesis’, high blood cholesterol is a major risk factor, while lowering cholesterol levels can reduce risk [5]. Dyslipidaemias (i.e., hypercholesterolaemia or hyperlipidaemia) are abnormalities of lipid metabolism characterised by increased circulating levels of serum total cholesterol, LDL cholesterol, triglycerides, and decreased levels of serum HDL cholesterol. High levels of LDL cholesterol and non-HDL cholesterol have been associated with cardiovascular risk, while other cholesterol-related serum markers, such as the small dense LDL cholesterol, lipoprotein(a), and HDL particle measurements, have been proposed as additional significant biomarkers for CVD risk factors to add to the standard lipid profile [6]. HDL cholesterol has been considered as the atheroprotective ‘good’ cholesterol because of its strong inverse correlation with the progression of CVD [7]; however, it is the functionality of HDL cholesterol, rather than its concentration that is more important for the preventative qualities of HDL cholesterol in CVD. In general, dyslipidaemias have been ranked as significant modifiable risk factors contributing to prevalence and severity of several chronic diseases including aging, hypertension, diabetes, and CVD. High serum levels of these lipids have been associated with an increased risk of developing atherosclerosis [8].

Furthermore, dyslipidaemias have been characterised by several studies not only as a risk factor but as a “well-established and prominent cause” of cardiovascular morbidity and mortality worldwide [9]. Even though such an extrapolation is not adequate, it was, however, not surprising that this was made, because since the term arteriosclerosis was first introduced by pioneering pathologists of the 19th century, it has long been believed that atherosclerosis merely involved the passive accumulation of cholesterol into the arterial walls for the formation of foam cells. This process was considered the hallmark of atherosclerotic lesions and subsequent CVD. Moreover, one-sided interpretations of several epidemiological studies, such as the Seven Countries Study (SCS), have highlighted outcomes that mostly concerned correlations between saturated fat intake, fasting blood cholesterol concentrations, and coronary heart disease mortality [10,11,12,13]. Such epidemiological correlations between dyslipidaemias and atherosclerosis led to the characterisation of atherosclerosis as primarily a lipid disorder, and the “lipid hypothesis” was formed, which would dominate thinking for much of the 20th century.

In the clinical setting, in order to address the lipid hypothesis, the levels of cholesterol related plasma lipoproteins and triglycerides (lipid profile) have been used as traditional biomarkers for cardiovascular risk, but also for dietary and treatment guideline designs [5]. Dietary and medical guidelines have focused on the reduction of cholesterol and lipid levels as the best way to prevent chronic diseases such as CVD [5,9]. Such guidelines suggest the application of statin therapies in order to reduce the levels of cholesterol (through inhibition of cholesterol synthesis by HMG-CoA reductase inhibitors); however, numerous side effects have been reported, including the development of other chronic diseases such as diabetes mellitus [14]. Moreover, specific dietary strategies for reducing cholesterol intake are the mainstay of management in most cases of dyslipidaemia, prior to, or simultaneously with, the initiation of a lipid lowering agent [9]. Dietary fats, cholesterol, and the levels of serum cholesterol in relation to the development of CVD have been somewhat demonised.

On the other hand, since cholesterol is an essential biomolecule for the normal function of all our cells, an emerging question has recently surfaced: “how much do we need to lower the levels of cholesterol”? Furthermore, given the fact that cholesterol plays a crucial role in several of our cellular and tissue mechanisms, it is not surprising that there are several consequences due to the aggressive reduction of cholesterol levels in the body, which has been common practice over the last few decades. In addition, targeting cholesterol and fat intake by introducing diets with low-fat products and by reducing the intake of high-fat foods can lead to less absorption and lower bioavailability of other lipids containing high value nutrients, such as several lipid soluble vitamins (especially vitamin D) and other lipid molecules. Such lipids have exhibited a plethora of beneficial bioactivities, not only related to reducing the risk of chronic diseases but also through a wide range of important bio-functionalities and anti-inflammatory properties [3]. Therefore, lower cholesterol levels do not equate to better health, or to lower risk of chronic diseases such as CVD. Homeostasis must be maintained, even with regard to cholesterol, both HDL and LDL [15].

Moreover, recent systematic reviews and meta-analyses have started to question the validity of the lipid hypothesis, as there is lack of an association or an inverse association between LDL cholesterol and both all-cause and CVD mortality in the elderly [15] and several cancers such as lung, prostate, and breast cancer [16,17,18]. Such studies provide the rationale for more research about the causes (and not only the risk factors) of chronic diseases such as atherosclerosis, CVD, and cancer, but also for a re-evaluation of the guidelines for cardiovascular prevention, in particular because the benefits of statin treatments have been exaggerated [15].

Statistical and epidemiological extrapolations often lack fully clarified biochemical mechanistic evidence, while associations and correlations do not necessarily mean causation. In addition, a follow-up by systematic reviews and meta-analyses often present contradictory outcomes against the initial results that were introduced by early stage epidemiological studies lacking consistency, biological gradient, and coherence. Thus, such extrapolations can lead to one-sided, premature targeting of risk factors accompanied with consequences, often without the desirable outcomes. Targeting a risk factor such as high serum cholesterol may decrease the probabilities for a disease, but usually cannot prevent the causation of chronic diseases.

### 1.3. Revisiting the Lipid Hypothesis: Outcomes of the Mediterranean Diet against Inflammation

Previous epidemiological and observational studies, such as the SCS in which the lipid hypothesis was mostly based, have been re-evaluated. For example, even though within the SCS the strength of the association between serum cholesterol and cardiovascular mortality were similar in different cultures, the absolute risks differed substantially. Kromhout reported that at a serum cholesterol level of 200 mg/dL, the 25-year cardiovascular mortality rate was five times higher in the Northern European populations of the SCS compared to the Southern Mediterranean populations [19], and thus the relations between diet, serum cholesterol, and cardiovascular mortality are more complex than originally thought. This is because it is not only dietary cholesterol involved, but other lipids and antioxidants may play a role in the onset and prevention of atherosclerosis [19]. Such a low prevalence of cardiovascular mortality in the Mediterranean cohorts of SCS is now attributed to their lifestyle and especially to their dietary habits, namely the traditional Mediterranean diet (Med-diet) [10,20]. A common feature of the diet amongst populations in the Mediterranean is a relatively high dietary intake of vegetables, fruits, legumes, whole grains, monounsaturated fats, and nuts, followed by moderate consumption of fish, dairy products (mainly cheese and yogurt), alcohol, and low consumption of red and processed meats [21].

The major outcomes of the SCS and other similar epidemiological studies (i.e., studies trying to decipher the ‘French Paradox’ [22]) concerning the protective effects of dietary patterns, such as the Med-diet against chronic diseases, were initially either neglected or misinterpreted. CVD and cardiovascular mortality occurred in much relatively lower rates in the Southern European populations (i.e., Italy and Greece) despite a rather high dietary intake of saturated fats and cholesterol [10,20,23]. A recent systematic review and meta-analysis revealed that Med-diet can actually reduce the incidence of cardiovascular events, breast cancer, and type II diabetes mellitus, without any restriction on fat intake [24].

Over the last 2 years there has been a significant number of studies referring to adoption of the Med-diet pattern and its associated beneficial outcomes in a plethora of several chronic diseases that are either directly or indirectly related to inflammation. These studies refer to heart failure, CVD [25], cancer [26,27], obesity [28], metabolic syndrome [29,30,31], diabetes [31,32,33,34], and other subsequent manifestations such as diabetic retinopathy [35], asthma [36], autoimmune diseases such as rheumatoid arthritis [37], incident frailty risk [38], non-alcoholic fatty liver disease [39,40], inflammatory bowel disease [41], cognitive health, the risk of Alzheimer’s disease and dementia [42,43,44], and age-related macular degeneration [45].

In addition, the Med-diet has also been associated with beneficial outcomes, even in secondary CVD prevention [46]. When patients suffering from CVD or diabetes follow the Mediterranean dietary pattern, the incidence of recurrent myocardial infarction and cerebrovascular events is reduced. The protective effect of this dietary pattern can be maintained for up to four years after the first infarction (Lyon Diet Heart Study) [47]. Moreover, in contrast to the contradictions of lipid hypothesis and mortality in elderly people [15], the HALE project has also shown that individuals aged 70 to 90 years following a Med-diet and healthy lifestyle have a 50% lower rate of all-cause and cause-specific mortality [48]. Followers of the Med-diet are also less likely to suffer sudden cardiac death and age-related cognitive decline [49].

The inverse association between Med-diet and all causes of diseases and cardiovascular-mortality has been attributed to several of its pleiotropic protective effects. For instance, the Med-diet can beneficially influence several risk factors such as lowering BMI, blood pressure, reducing insulin resistance, reducing lipid levels (i.e., the ratio of cholesterol/HDL cholesterol), and improving HDL-cholesterol functionality [50,51,52,53,54]. However, the main beneficial impact of Med-diet is on the improvement of endothelial function and the decrease of the inflammatory milieu, inflammation-related mediators, biomarkers such as platelet-activating factor (PAF), and several cytokines. It is also suggested that there is an improvement of oxidative stress, with lower concentrations of oxidised LDL and improved apolipoprotein profiles, and, finally, there is evidence of beneficial effects against platelet aggregation and blood coagulation [3,55,56,57,58].

The overall outcomes and beneficial effects of Med-diet have radically shifted the attention from the lipid-centric model that is characterised by the desired reduction of cholesterol levels to more effective targeting against the factual causative factors of chronic diseases, which are inflammation and its related manifestations. Prevention is key to reducing global mortality due to chronic diseases such as CVD; therefore, it is imperative to separate the underlying causes and processes of the disease from the risk factors and symptoms of disease. The clarification of the key roles and interplay of various cells, inflammatory mediators, and pathways during chronic inflammatory manifestations related to the onset of several chronic diseases is of great importance and may lead to a plethora of novel potential targets for fine-tuning of the inflammatory response during the chronic smouldering of inflammation that characterises these disorders.

## 2. Re-Discovering Chronic Inflammation as the Cause for Chronic Diseases

Inflammation is a physiological reaction of the innate immune system that maintains a constant internal milieu while being exposed to continuously changing environmental pressures, irrespective of whether the initial causes originate from mechanical, physical, chemical, infectious, immunological, or reactive natural traumatic injury or metabolic dysfunction. The inflammatory response aims to reduce the agent that causes tissue injury and/or minimise these effects, to induce appropriate wound healing and to restore tissue homeostasis. Inflammatory responses are initiated by innate sensing mechanisms that detect the presence of microbial infection, stressed or dying cells, loss of cellular integrity, barrier breach, etc. A cascade of inflammatory pathways and mechanistic effects is supposedly well-orchestrated by the immune system in order to eradicate the causative agent.

Several immune cells can change their number, morphology, and nature depending on the stage and type of inflammation. Biochemically, inflammation is denoted by a local increase of numerous tissue hormones, transmitters, complement components, cytokines, and lipid mediators such as PAF and eicosanoids. Most of these products are autacoids that are synthesised at the site of inflammation in order to resolve the inflammatory process by removing or inhibiting the actions of the triggering agent [8]. Provided that the immune response succeeds in eliminating the infectious agent or to repair the initial tissue injury, the inflammatory process will be terminated in a timely fashion and thus only affects tissue function transiently.

However, in cases where the inflammation fails to resolve due to the persistence of the triggering agent or due to unsuccessful repair of the initial tissue injury or dysfunction, a sustained underlying inflammatory process develops, leading to further tissue dysfunction and detrimental consequences. Several traditional and emerging risk factors are thought to influence our health and, especially, inflammation-related chronic diseases, by their interrelation with underlying molecular and cellular manifestations that result in chronic inflammatory responses leading to the loss of tissue homoeostasis and dysfunction. Apart from dyslipidaemias, other well-established risk factors include hypertension, diabetes, smoking, excessive food intake, previous infections (influenza, oral pathogens) or underlying autoimmune diseases such as lupus or rheumatoid arthritis, pollution, and genetic abnormalities [59]. It is now well established that a common junction of such risk factors is chronic and unresolved inflammatory manifestations. Inflammation that causes endothelial dysfunction seems to be the key causative underlying mechanistic player, at the molecular and cellular level, for the onset and development of subsequent inflammation-related chronic disorders such as atherosclerosis and subsequent CVD, ischemic and renal disorders, cancer metastasis, diabetes, infections, and comorbidities [8,57,58,59,60,61,62].

For example, in cases of dyslipidaemia, increased cholesterol levels are not the causative agent or the underlying biochemical mechanism responsible for endothelial dysfunction and atherosclerosis development. The accumulation of excess plasma LDL cholesterol is addressed by the innate immune system as an undesired event. Therefore, an inflammatory response at the endothelial wall is promoted to reduce the threat by the removal of excess LDL and oxidised-LDL (Ox-LDL) cholesterol from the blood stream to the subendothelium, where they are engulfed by comigrated monocytes for final removal [63,64]. During chronic inflammatory diseases, inflammation and infections can also induce a variety of alterations in lipid metabolism, including decreases in serum HDL cholesterol, increases in triglycerides, lipoprotein(a), and LDL levels. These changes of the lipid levels may initially dampen inflammation or fight infection; however, the sustained inflammation can contribute to the increased risk of atherosclerosis [65]. In addition to affecting serum lipid levels, inflammation also adversely effects lipoprotein function; LDL is more easily oxidised, as the ability of HDL to prevent the oxidation of LDL is diminished, while several steps in the reverse cholesterol transport pathway are also adversely affected during inflammation. The greater the severity of the underlying inflammatory disease, the more consistently these abnormalities in lipids and lipoproteins are observed [65]. Thus, it is not serum cholesterol and lipoproteins that influence the endothelium but the inflammatory response that affects the well integrity and functionality of the endothelium.

Apart from the effects of inflammation on plasma lipids, it is now well established that more important soluble and cellular immune factors associated with chronic inflammation can promote inflammation-related endothelial dysfunction and atherogenesis, either during dyslipidaemia or independently of dyslipidaemia [66]. Even though atherosclerosis and CVD were previously viewed as lipid storage disorders, we now recognise that inflammation drives much of endothelial dysfunction and mechanisms of clinical complications with these diseases and related comorbidities, such as sepsis [67,68], human immunodeficiency virus (HIV) infection [69,70,71,72,73,74], periodontal diseases [75,76,77], kidney disorders [78,79,80,81], healthy ageing, and inflammatory autoimmune diseases such as systemic lupus erythematosus and rheumatoid arthritis, independently of traditional cardiovascular risk factors such as serum lipid levels [66,82,83].

Inflammation plays a key role in all stages of the formation of vascular lesions maintained and exacerbated by several risk factors such as unhealthy diet and lifestyle, smoking, hyperlipidaemia/hypercholesterolaemia, hypertension, autoimmune diseases, etc. The consequence of chronic inflammation is endothelial dysfunction that sets in, and we can define it as an integrated marker of the damage to arterial walls by classic risk factors. Endothelial dysfunction is usually characterised by an inflammation-related milieu acting on leukocytes and endothelial cells, through an interplay with other immune cells such as T lymphocytes, mast cells, dendritic cells (DC), and platelets [57,58,66,84,85]. The orchestrated overexpression and increased production of pro-inflammatory cytokines occurs, including interleukin-6 (IL-6), tumour necrosis factor (TNF) and its receptor, high-sensitivity C-reactive protein (hsCRP), type I interferons (IFN-α, IFN-β), adhesion molecules, chemokines, and lipid inflammatory mediators such as PAF and eicosanoids. Other linked events include the increased generation of reactive oxygen species (ROS), the increased oxidation of LDL cholesterol, and the reduction of protective nitric oxide levels.

Therefore, the mechanistic pathways and key players implicated in the inflammatory crosstalk taking place throughout the onset, development, and progression of chronic diseases is of great importance, in order to unravel putative preventive and therapeutic targets with less side effects. The inverse effects of the Med-diet with chronic diseases is mostly related to the pleiotropic effects and interplay of its food constituents on all these inflammation-related pathways; following a Mediterranean dietary pattern leads to the reduction of several inflammatory mediators and biomarkers related to the endothelial functionality, such as decreases in hsCRP, IL-6, and intracellular adhesion molecule-1 (ICAM-1) [27].

## 3. The Role of PAF in Chronic Diseases and the Beneficial Effects of the Mediterranean Diet

### 3.1. PAF Structure, Activities, and Metabolism: The Role of PAF

#### 3.1.1. PAF Structure and Physiological Roles

**Figure 1:**
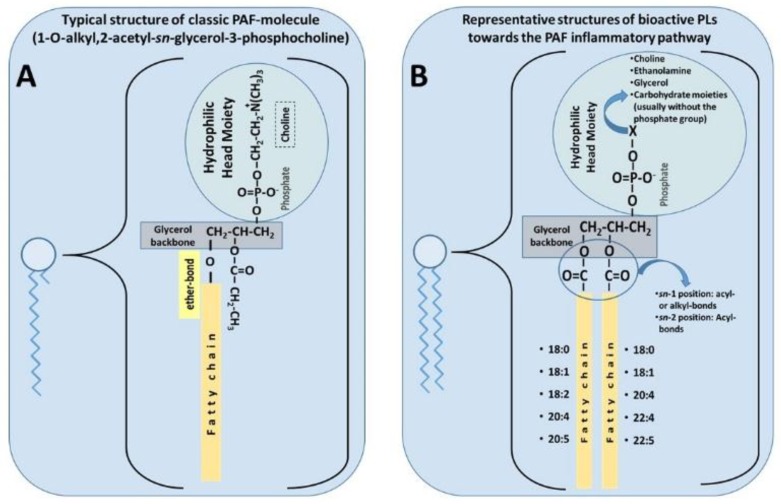
(**A**) Typical structure of classic platelet-activating factor (PAF) molecule [86]. (**B**) Representative structures of bioactive polar lipids (PL) towards the PAF inflammatory pathways (**B**), which have been identified in several foods of the Mediterranean diet [56].

PAF is a potent lipid inflammatory mediator with pleiotropic effects that are implicated in several chronic diseases [57]. The classic PAF molecule is characterised by an alkyl ether linkage at the *sn*-1 position, an acetyl group at the *sn*-2 position, and a phosphocholine group at the *sn*-3 position of glycerol backbone (1-O-alkyl-2-*sn*-acetyl-glycero-3-phosphocholine, [86]) (Figure 1A). These three structural features are all equally important requisites for the optimal biological activity of PAF, mediated by its stereospecific binding to its specific receptor [87,88]. Because of the ether linkage at the *sn*-1 position, the classic molecule of PAF is an unusual lipid, as such moieties are not common in animals, nor is it common to find the acetic acid esterified directly to glycerol at the *sn*-2 position. Thus, it seems that PAF was chosen by evolution to participate in specific functions in several of our cells, tissues, organs, and throughout the body. PAF was the first intact phospholipid known to have messenger functions by binding to a specific receptor on the cell membrane, and not simply via physicochemical effects on the plasma membrane of the target cell [79].

Lately, the term ‘PAF family’ has been proposed to include every other phospholipid molecule called PAF-like molecules, which have similar structures to those of the classic PAF molecule, and they exhibit similar bioactivities [89]. However, such PAF-like moieties are usually less potent than PAF by several orders of magnitude, i.e., increasing the chain length beyond 3 carbons at the *sn*-2 position decreases its biological potency; likewise, altering the polar group at *sn*-3 position decreases the potency of the molecule. The molecular composition of PAF varies depending on different species and cell types. Related PAF-like lipids include, for example, the acyl-phosphatidylcholine-PAFs (with a short chain acyl group at the *sn*-2 position), ethanolamine-PAFs, inositol-PAFs, oxidised alkyl-acyl phosphatidyl glycerophosphocholines [90,91], and hydroxyl-alkyl acyl phospholipids [76,77].

PAF, in general, play a vital role in various physiological processes such as mediation of normal inflammatory responses, regulation of blood circulation and pressure, regulation of coagulation responses, glycogen degradation, brain function, reproduction, foetal implantation, lung maturation, initiation of parturition, and exocrine gland functions [92]. However, PAF can be regarded as both a friend, since it is presumed to have evolved as part of a protective mechanism in the innate host defence system, but also as a foe, because of its involvement in uncontrolled inflammation-related pathological conditions [93]. When present in excess, PAF has been implicated in the pathogenesis of several inflammation-related chronic disorders [57]; thus, its synthesis, distribution, and degradation are all strictly controlled, as would be predictable for such a potent molecule with a wide range of diverse actions.

#### 3.1.2. The PAF/PAF-Receptor Signalling Pathways

PAF and PAF-like molecules act through their binding to a unique G-protein coupled seven transmembrane receptors, called the PAF-receptor (PAF-R) [87,88]. Species identity, differentiated by heterogeneity in linkage, degree of unsaturation, and carbon chain length of the alkyl or acyl chains at the *sn*-1 and *sn*-2 position, partially dictates signalling specificity by eliciting various signal transduction pathways following PAF-R activation [94,95]. The PAF-R is constitutively present on platelets, leukocytes, and endothelial cells, and further expression may be induced by appropriate stimuli. PAF-R is highly expressed by cells within the innate immune and cardiovascular systems [96], pointing to a role for PAF and PAF-like molecules as pleiotropic communicators in plasma [97].

**Figure 2:**
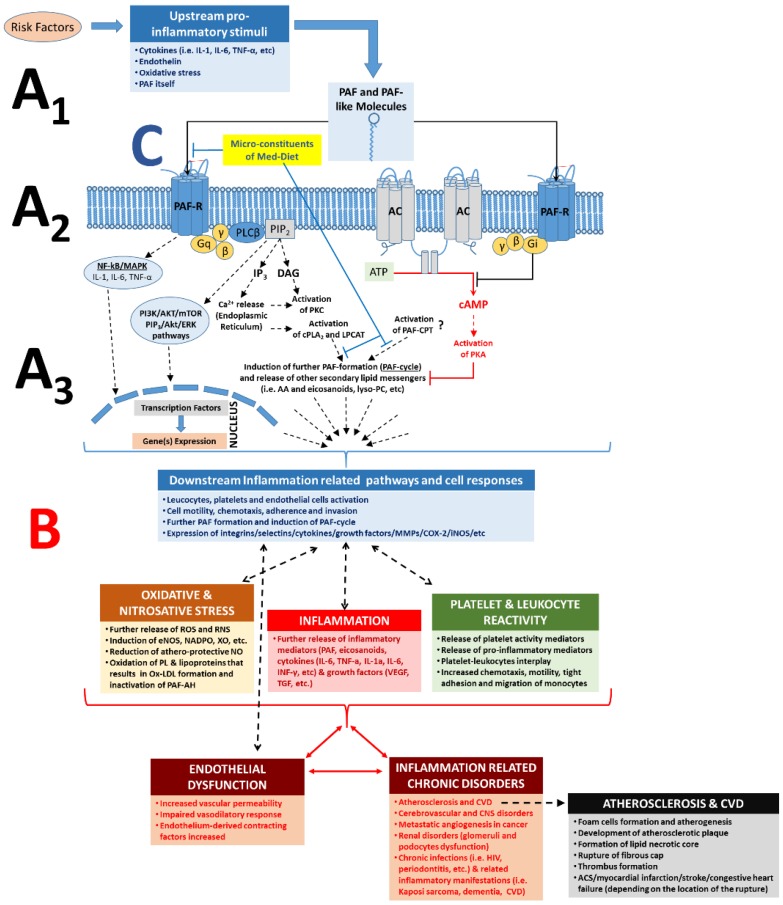
Role of PAF, PAF-R, and its related pathways in the inflammatory cascades and in the pathogenesis of inflammation-related chronic disorders; increased PAF levels by pro-inflammatory stimuli and binding of PAF on its receptor, PAF-R, on the membranes of several cell types can lead to intracellular cascades and a PAF cycle-related amplification of the initial stimuli (**A**) and in numerous cell responses according to each cell type (**B**), which can lead to endothelial dysfunction and the onset and progression of inflammation-related chronic diseases. A1. Several risk factors and related upstream pro-inflammatory stimuli trigger formation of PAF and PAF-like molecules (i.e., oxidised phospholipids) and expression of PAF-R. A2. Binding of PAF/PAF-like molecules on PAF-R promote several inflammation-related intracellular pathways; activation of the PAF-R signalling initiates (through a Gq-linked mechanism) PLCβ-mediated hydrolysis of PIP_2_ to produce IP_3_ and DAG, leading to transient elevation of cytosolic Ca^2+^ released from intracellular stores and activation of PKC. The rise in Ca^2+^ also activates cPLA_2α_, leading to the release of AA and lysophosphatides, which can serve as substrates for further synthesis of eicosanoids and PAF, respectively. Signalling through Gi-linked PAF-R inhibits the conversion of ATP to cAMP by adenylate cyclase, in this way preventing the activation of PKA and related anti-inflammatory signalling events. A3. Activation of the PAF/PAF-R intracellular pathways leads to the activation of cPLA_2_ and PAF biosynthetic enzymes (LPCAT) for further formation of PAF and other lipid second messengers, thus creating a PAF cycle and further amplification of the initial inflammatory stimuli, while expression of genes involved in inflammatory manifestations (such as genes of several cytokines, integrins, selectins, metalloproteinase, several enzymes for eicosanoids, and ROS, etc.) is also induced. The pathways inducing the PAF-CPT-related synthesis of PAF are not fully elucidated. B. Increased PAF levels at the site of inflammation and ligand binding (PAF and/or oxidised phospholipids binding) on PAF-R can promote a broad spectrum of PAF effects depending on the cell type and tissue, which is achieved through the production and release of various downstream mediators, such as PAF itself and several other mediators of inflammation such as eicosanoids, cytokines (i.e., TNF-α, IL-1α, IL-6, IL-8, INF-γ, etc.), growth factors (i.e., VEGF, IGF, TGF), ROS, and RNS, but also through the expression of selectins and integrins (i.e., ICAM, VCAM, P-Selectin, E-Selectin) in the membranes of activated cells. Thus, increased downstream mediators, PAF levels, and the subsequent further activation of the PAF/PAF-R pathways promotes the activation and aggregation of platelets and leukocytes, activation of endothelial cells, leukocyte adherence, motility, chemotaxis, invasion, migration, and subsequent endothelial dysfunction, thus stimulating the onset and development of inflammation-related chronic diseases and disorders. C. Microconstituents of several foods of the Mediterranean diet have been found to beneficially inhibit the PAF/PAF-R pathways and PAF synthesis towards homeostatic re-equilibration of PAF levels and activities [57]. PAF: platelet-activating factor; PAF-R: G-protein-coupled PAF-receptor; AC: adenylate cyclase; NF-kB: nuclear factor-kappa light-chain-enhancer of activated B cells; MAPK: mitogen activated protein kinase; ERK: extracellular signal-regulated kinases; Akt: protein kinase B; PI3K: phosphatidylinositol 3-kinase; mTOR: mechanistic target of rapamycin; DAG: diacylglycerol; AA: arachidonic acid; cPLA_2_: cytosolic phospholipase A_2_; PKC: protein kinase C; PKA: protein kinase A; LPCAT: acetyl-CoA: lyso-PAF acetyltransferases; PAF-CPT: dithiothreitol l-insensitive CDP-choline: 1-alkyl-2-acetyl-*sn*-glycerol cholinephosphotransferase; ATP: adenosine triphosphoric acid; cAMP: cyclic adenosine monophosphate; PLC: phospholipase C; MMP: metalloproteinase; COX: cyclooxygenase; iNOS: nitric oxide synthase; eNOS: endothelial nitric oxide synthase; ROS: reactive oxygen species; RNS: reactive nitrogen species; NADPO: nicotinamide-adenine dinucleotide phosphate oxidase; XO: xanthine oxidase; IL-6: interleukin-6; IL-1: interleukin-1; TNFα: tumour necrosis factor-α; ACS: acute coronary syndrome; VEGF: vascular endothelial growth factor; PL: phospholipids; CVD: cardiovascular diseases; CNS: central nervous system.

Ligand binding (PAF and/or PAF-like molecules) to the PAF-R subsequently triggers multiple intracellular signalling pathways and gene-expressions, depending on the target cell and PAF levels (concentration) in blood or tissue [87,88,89,98] (Figure 2 (A1–A3)). For example, activation of the PAF-R signalling initiates (through a Gq-linked mechanism) PLCβ-mediated hydrolysis of PIP2 to produce IP3 and DAG, leading to transient elevation of cytosolic Ca^2+^ released from intracellular stores and activation of PKC. The rise in Ca^2+^ also activates cPLA2α, leading to the release of arachidonic acid (AA) and lysophosphatides, which can serve as substrates for further synthesis of eicosanoids and PAF, respectively. In addition, signalling through Gi-linked PAF-R inhibits the conversion of ATP to cAMP by adenylate cyclase, thus preventing the activation of PKA and related signalling events.

Signalling through other pathways is also amplified by the PAF/PAF-R pathway activation, since inhibition of PAF synthesis or PAF-R blockade significantly attenuates signalling through apparently unrelated pathways, suggesting a critical role for PAF/PAF-R action as a co-stimulatory signal. For example, many VEGF-directed effects on vascular endothelium require PAF synthesis [57]. Nevertheless, the activation of the PAF/PAF-R pathway further triggers the activation and aggregation of platelets and leukocytes and promotes leukocyte and platelet adherence, motility, chemotaxis, invasion, migration, ROS generation, and further PAF formation (Figure 2) [89,98].

#### 3.1.3. PAF Levels Result from Enzymatic Biosynthesis, Non-Enzymatic Oxidative Synthesis, and Enzymatic Catabolism

**Figure 3:**
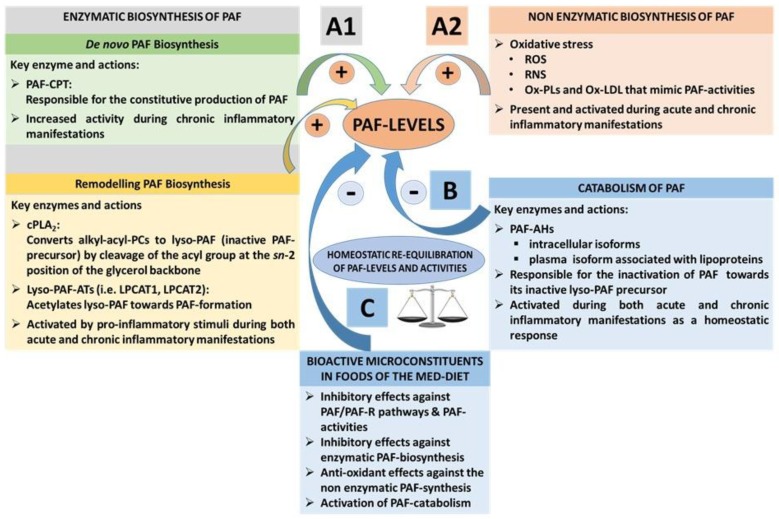
PAF levels result from enzymatic biosynthesis, non-enzymatic oxidative synthesis, and enzymatic catabolism, while bioactive microconstituents of the Med-diet beneficially affect these pathways. (**A1**) The enzymatic biosynthesis of PAF contributes to basal PAF levels or a periodic increase of PAF levels during normal inflammatory responses, while during unresolved and chronic inflammatory manifestations, the enzymatic biosynthesis of PAF is responsible for pathologically increased PAF levels through a continuous induction of the PAF cycle; (**A2**) Non-enzymatic synthesis of PAF occurs during oxidative stress, increasing ROS and RNS and inducing the synthesis of PAF and PAF-like molecules. When Ox-LDL is produced, PAF-like molecules mimic the activities of PAF. These pathways are not regulated enzymatically; (**B**) Catabolism of PAF is enzymatically regulated by PAF-AH. PAF catabolism is activated during both acute and chronic inflammatory manifestations and inactivates both PAF and PAF-like molecules; (**C**) Bioactive microconstituents present in foods of the Med-diet (i.e., polar lipids) have demonstrated beneficial outcomes by inducing homeostatic equilibration of PAF levels and activities through the Inhibition of the PAF/PAF-R pathways and modulation of the PAF anabolic and catabolic enzymes. PAF: platelet-activating factor; PAF-R: G-protein coupled PAF-receptor; PAF-CPT: dithiothreitol l-insensitive CDP-choline: 1-alkyl-2-acetyl-*sn*-glycerol cholinephosphotransferase; Lyso-PAF-ATs (LPCAT1, LPCAT2): acetyl-CoA: lyso-PAF acetyltransferases; cPLA_2_: cytoplasmic phospholipase A_2_; PAF-AH: PAF-acetylhydrolase; PC: Phosphatidylcholine; ROS: reactive oxygen species; RNS: reactive nitrogen species; LDL: low-density lipoprotein; Ox-LDL: oxidised-LDL; Med-diet: Mediterranean diet.

Under normal circumstances, homeostatic levels of PAF present in plasma and biological tissue seem to be regulated by a balance of its biosynthetic and catabolic enzymatic pathways [57]. PAF is synthesised throughout the body by the specific stimulation of various cell types such as platelets, macrophages, monocytes, eosinophils, basophils, and endothelial cells. PAF is mostly produced in the blood, lungs, kidney, myocardium, brain, liver, skin, saliva, retina, uterus, and embryo [56,99,100]. Two enzymatic pathways by which PAF is biosynthesised in the body are the ‘remodelling’ and the ‘*de novo*’ pathways (Figure 3(A1)).

The remodelling enzymatic pathway of PAF biosynthesis involves remodelling of a membrane lipid constituent (a long-chain fatty acyl residue in *sn*-2 is replaced with an acetyl residue), and it has been proposed that this pathway is periodically involved in the acute pro-inflammatory production of PAF under activation of several cells during inflammation [101]. More specifically, the action of cytoplasmic phospholipase A_2_ (PLA_2_) yields a precursor of PAF called lyso-PAF (1-O-alkyl-*sn-*glyceryl-3-phosphorylcholine), which is then acetylated by at least two isoforms of acetyl-CoA: lyso-PAF acetyltransferases, namely, LPCAT1 and LPCAT2 (lyso-PAF AT), leading to the formation of PAF [102]. LPCAT2 is highly expressed in inflammatory cells, and, depending upon the inflammatory stimulus used to activate the cells, PAF is produced within seconds, minutes, or hours following stimulation. In addition, PAF itself can act as an inflammatory signal, and the binding of PAF to its receptor on inflammatory cells can promote the very rapid (within 30 s) production of PAF; PAF-induced, protein kinase, Cα-mediated phosphorylation of LPCAT2 enhances enzymatic activity, leading to the vary rapid production of PAF. Thus, a PAF cycle can consistently induce increased PAF levels and subsequent inflammatory cascades (Figure 2 and Figure 3)

The *de novo* enzymatic pathway of PAF biosynthesis is similar but distinct to the biosynthesis of phosphatidylcholine, since a phosphocholine function is transferred to alkyl acetyl glycerol. This pathway has been initially reported as the pathway responsible for the constitutive production of PAF basal levels. A key step in this route is the conversion of 1-O-alkyl-2-*sn*-acetyl-glycerol to PAF by a specific dithiothreitol l-insensitive CDP-choline: 1-alkyl-2-acetyl-*sn*-glycerol cholinephosphotransferase (PAF-CPT) [57,81]. Interestingly, apart from the remodelling pathway, which is always activated in both acute and chronic inflammation, the key enzyme of the ‘*de novo* pathway, PAF-CPT, seems to be more active during chronic inflammatory manifestations, thus contributing to an increase of basal levels of PAF that seem to be related to the continuous activation of inflammatory cascades in the long-term during the development of inflammation-related chronic disorders [57,70,81]. Thus, the regulation of the biosynthetic pathways of PAF seems to be more complicated than was initially thought, while both PAF biosynthetic routes are correlated with well-established inflammatory and immunological biomarkers (i.e., several cytokines, viral load, CD-40L, etc.) in several cases [57,69,70,79,81,103,104].

Apart from its enzymatic biosynthetic pathways, PAF and PAF-like lipids can also be produced through non-enzymatic synthesis by oxidation of other lipids during oxidative stress [105,106]. The production of PAF and such PAF-like oxidised lipids usually occurs during inflammation and oxidative stress (Figure 3(A2)). Vice versa, PAF and PAF-like lipids can also stimulate the production of ROS and nitrogenous species such as reactive nitrogen species (RNS) during oxidative and nitrosative stress in inflammation-induced endothelial dysfunction and atherosclerosis [89].

The main catabolic enzyme that reduces PAF levels is PAF acetylhydrolase (PAF-AH), delicate phospholipase A_2_ that removes the acetate group from the PAF molecule and thus transforms PAF to its inactive form of lyso-PAF (Figure 3B) [107]. These enzymes, PAF-AH, are produced largely by hepatocytes and macrophages, and are widely distributed in human plasma, blood cells, and a variety of tissues. Subsequent research revealed that the PAF-AH family includes intracellular forms called PAF-AH I and PAF-AH II, as well as an extracellular third isoform [108]. PAF-AH, an extracellular isoform in plasma, is a member of the PLA_2_ superfamily of enzymes that is also known as lipoprotein-associated phospholipase A_2_ (Lp-PLA_2_), since it circulates in blood in association with plasma lipoprotein particles such as LDL and HDL, or the PLA_2_ group 7 (PLA_2_G7) [107,108,109,110]. Intracellular PAF-AH type I exists in the cytoplasm of many (probably all) types of mammalian cells and tissues [111]. Interestingly, the intracellular PAF-AH Type II that has no homology with PAF-AH I, but shares sequence similarity to plasma PAF-AH, was reported to act as a cellular Phospholipase A_2_ that hydrolyses oxidatively modulated or truncated phospholipids (with short length or oxidatively modified *sn*-2 acyl chains). It is thus suggested that PAF-AH (II) functions as an antioxidant phospholipase that plays a protective role also against oxidative stress [108,112].

### 3.2. The PAF Pathway and Metabolism in Chronic Diseases

Under normal conditions, plasma and tissue levels of PAF are tightly regulated by its metabolic pathways. However, production of PAF and PAF-like molecules can become elevated and/or dysregulated during extended periods of immune activation and chronic inflammation-related disorders by amplification of its synthesis, either through cascades activating its biosynthetic enzymes or through oxidative production of PAF, or usually by both [57,69,70,79,81,103,104,113]. PAF plays a major role in the physiopathology of inflammatory reactions and is produced and released in large quantities by inflammatory cells in response to specific stimuli, such as upstream regulators (IL-1, IL-6, TNF-α, Endothelin, oxidative stress, and PAF itself; Figure 2A) [57,78,89,114].

Increased PAF levels at the site of inflammation can activate several cell types through its receptor. This leads to the initiation of a broad spectrum of PAF effects depending on the cell type and tissue, which is achieved through the production and release of various downstream mediators, such as PAF itself and several other mediators of inflammation such as eicosanoids, cytokines (i.e., TNF-α, IL-1α, IL-6, IL-8, INF-γ, etc.), growth factors (i.e., VEGF, IGF, TGF), ROS, and RNS, but also through the expression of selectins and integrins (i.e., ICAM, VCAM, P-Selectin, E-Selectin) in the membranes of activated cells (Figure 2B) [57,58,78,89,113,114].

The interconnected crosstalk between PAF, pro-inflammatory upstream mediators that induce PAF production, and PAF-induced downstream mediators seems to be interrelated during inflammatory manifestations and inflammation-related chronic diseases. These pathways serve as one of the main junctions between many inflammatory cascades that ultimately lead to endothelium dysfunction and inflammation-related disorders such as atherosclerosis, CVD, renal disorders, cerebrovascular, central nervous system (CNS) disorders, metastatic angiogenesis during cancer, sepsis, and several other chronic disorders (Figure 2B) [57,58,78,89,113].

#### 3.2.1. PAF in Atherosclerosis and CVD

Cardiovascular diseases (CVD) are the leading cause of death worldwide. It is estimated that 49 million people are now living with the disease in the European Union alone [115]. Atherosclerosis is a slow progressive disease in which lesions or plaques form in large and medium-sized arteries, consisting of necrotic cores, calcified regions, accumulated modified lipids, migrated smooth muscle cells (SMC), foam cells, endothelial cells, and several leukocyte subtypes. Monocytes, circulating blood precursors of tissue macrophages, and myeloid-derived DC influence plaque development following recruitment into the intima and differentiation to foam cells.

In contrast to the previous notions concerning the passive accumulation of lipids in macrophages during the formation of foam cells, it is now clear that there are more complex inflammatory mechanisms acting on monocytes, macrophages, platelets, several other leucocyte subtypes, and endothelial cells that seem to promote atherosclerosis via pro-inflammatory foam cell formation [66]. Persistent and unresolved inflammation at the vascular wall gives rise to inappropriate platelet and leukocyte recruitment at the endothelium. The inflammatory interplay and crosstalk between these cells and endothelial cells, facilitated by several inflammatory mediators, initiates the cascades that induce chronic inflammatory manifestations at the vascular wall, which counteracts the homeostatic inflammatory response, leading to endothelial dysfunction and initiation of proatherogenic events that lead to atherogenesis and atherosclerosis [116]. PAF is one of the main junctions between several inflammatory pathways (cytokines, oxidative stress, eicosanoids, etc.) and their interplay with cells participating in inflammation-related atherosclerosis. Therefore, PAF is implicated in all stages of atherosclerosis, from the initiation of atherogenesis all the way through to plaque formation, development, instability, and rupture [58,89,105,117].

##### The Pro-Inflammatory Crosstalk between PAF with Several Cells and the Endothelium Induces Early Pro-Atherogenic Phases of Endothelial Dysfunction

At early pro-atherogenic conditions, PAF is produced in several cells, such as platelets, leukocytes, and endothelial cells under pro-inflammatory stimuli and/or by the oxidation of lipoproteins. Thus, PAF can further propagate oxidative stress, through the oxidation of LDL and the reduction of NO bioavailability, but mostly by acting as a potent chemotactic factor for other human cells that exhibit its receptor on their membranes, such as monocytic and granulocytic leukocytes of the innate and adaptive immune system, endothelial cells, etc. Following these activations, a number of mediators are released by these activated cells (e.g., PAF itself, several cytokines, eicosanoids, ROS, RNS, and several enzymes), while adhesive molecules are expressed in their cell membranes (i.e., chemokines, selectins, and integrins, such as E-selectin, P-selectin, MCP1, ICAM-1, VCAM-1, etc.) that facilitate platelet-platelet, platelet-leukocyte, and platelet-leukocyte-endothelium aggregates and interplay [58,89]. The PAF pathway downstream products can further contribute to the propagation of atherosclerosis.

Molecules of the selectin family mediate interactions between platelets and leukocytes, with the endothelium allowing leukocytes and platelets to roll along the vascular endothelium wall. Platelet binding of the endothelium seems to precede the appearance of leukocytes in plaques and induces bidirectional expression of adhesion molecules and the production of monocyte attracting chemokines, such as PAF that plays a central role in cytokine-induced monocyte adherence to endothelium [58,89,117,118]. Activated platelets that adhere to the inflamed endothelium may enhance leukocyte recruitment, activation, and transmigration, thereby enhancing the inflammatory processes underlying atherosclerosis [119]. PAF and Leukotriene B4 (LTB4), derived by activated platelets, leukocytes or endothelium, but also thrombin (through PAF and LTB4 pathways), can propagate the activation of platelets and the subsequent activation and adhesion of leucocytes through the interplay of chemokines and their receptors [117]. An important aspect of this platelet-leucocyte interplay is the diversity of leukocytes recruited by vessel wall adherent platelets, such as the platelet-mediated recruitment of neutrophils, monocytes, DC, T-lymphocytes, B-lymphocytes, and NK-cells to endothelium [117].

In addition, platelets regulate neutrophil activation through the generation of PAF as a chemoattractant pro-inflammatory lipid [120]. Activated endothelial cells and platelets generate considerable amounts of PAF, which act cooperatively with other extracellular stimuli to induce full integrin activation and leukocyte arrest [58,89,120]. However, whether PAF mostly originates from activated platelets, endothelial cells or leukocytes are not well defined yet [120]. Independently of its origin, the presence of PAF activates through its PAF/PAF-R pathways expression of integrin molecules at cell membranes to promote firm adhesion between leukocytes, platelets, and vascular endothelium [117].

PAF, other vasoactive compounds, angiogenic compounds, and pro-inflammatory mediators, such as arachidonic acid metabolites, histamine, cytokines, chemokines, and proteolytic enzymes, can also be released by mast cells that accumulate in the human arterial intima and adventitia during atherosclerotic plaque progression, and thus aggravate atherogenesis [8]. Cytokines produced by mast cells may be activated by pro-inflammatory stimuli, including cytokines, hypercholesterolemia, and hyperglycaemia, and trigger the endothelial expression of adhesion molecules such as P-selectin, VCAM-1, and chemokines such as PAF that mediate the recruitment and adhesion of leukocytes [8].

Similar to other chemoattractants, PAF has been detected in circulation; however, this molecule is mostly cell membrane-associated and operates in a paracrine manner on the G-protein coupled receptors of neighbouring cells [58,89,120]. Thus, PAF is also a main player in juxtacrine signalling and adhesion of leukocytes to other cells, and has also been shown to regulate firm neutrophil adhesion on the surface of immobilised spread platelets [119,121]. The level of platelet stimulation impacts directly on neutrophil adhesion to platelets monolayer, upon which neutrophil activity is spatially regulated by PAF generation [58,89,120]. Platelets and activated neutrophils act jointly to induce expression of adhesion molecules, permeability changes, and limit the bioavailability of nitric oxide, altogether aggravating endothelial dysfunction and facilitating subsequent monocyte plaque recruitment [122].

##### The Inflammatory Crosstalk Between PAF and Several Cells at the Intima and Subintima Leads to the Induction of Plaque Development and Increased Plaque Growth and Expansion

In the aortic lumen, endothelial cells have been activated by the aforementioned PAF-implicated downstream manifestations, leading to increased endothelium permeability and endothelial dysfunction. Subsequent abnormal recruitment, migration, and infiltration of monocytes then take place in the intima and subintima. Within the intima, monocytes secrete lipoprotein-binding proteoglycans, resulting in increased accumulation of modified LDL, which sustains inflammation. In addition, once in the intima, differentiation factors such as the macrophage colony-stimulating factor (M-CSF) differentiate pro-inflammatory monocytes into inflammatory type macrophages that ingest modified lipoprotein to become foam cells [59,123].

Emerging evidence suggests that the role of monocytes and macrophages in atherosclerosis is not simply that of a passive acceptor of lipids [66]. Apart from their phagocytic roles, macrophages can also instruct or be instructed by other immune cells by producing various immune effector molecules and by acting as antigen-presenting cells (APC). Plaque-related macrophages can have many phenotypes and functions depending on the stage of the disease; several monocyte subtypes exist, and subsequently several pro-inflammatory and anti-inflammatory macrophage subtypes also exist, while macrophages can rapidly adapt their phenotype and consequently their function in response to changes of the microenvironment and intracellular signalling pathways [122]. After appropriate activation, macrophages can exhibit a pro-inflammatory phenotype that can further activate endothelial cells, which in turn triggers further blood monocyte recruitment [122,124]. Thus, upon activation, the pro-inflammatory subtype of macrophages and foam cells produce inflammatory cytokines and chemokines that enhance inflammation and further regulate monocyte and T cell infiltration [59,124].

Macrophages express a myriad of receptors including G-protein coupled receptors such as PAF-R, through which they scan their environment for activation or polarisation signals, e.g., cytokines, growth factors, oxidised phospholipids, etc., [59,124,125,126], while, when in the atherosclerotic plaque, macrophages are capable of releasing a large repertoire of pro-inflammatory cytokines according to their phenotype and depending on the plaque microenvironment, including IL-1, IL-6, IL-12, IL-15, IL-18, TNF family members, and PAF, as well as anti-inflammatory cytokines like IL-10 and TGF-β family members (TGF-β1, BMPs, GDFs) [58,59,124].

**Figure 4:**
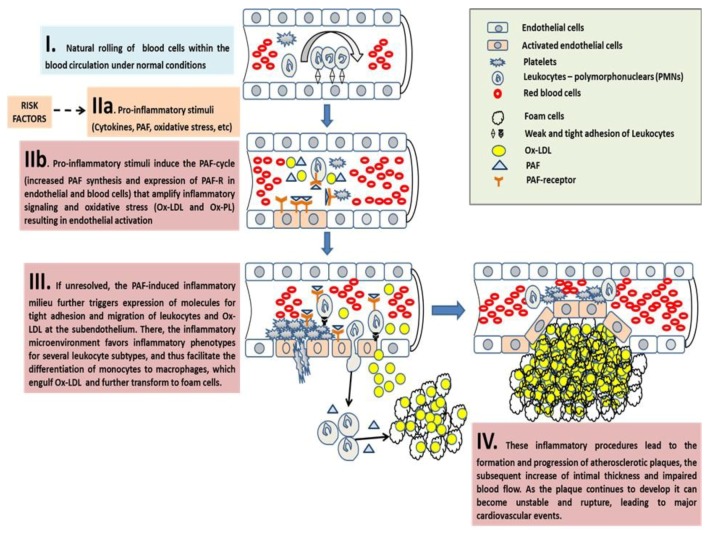
A schematic of the key role of PAF in the onset, progression, and expansion of atherosclerotic plaques and their subsequent cardiovascular disorders. Atherosclerotic events take place in four discrete stages (**IIa**–**IV**) as follows: (**I**) Under normal conditions, blood cells roll within the blood stream during physiological blood circulation. Leukocytes scavenge the endothelium by weak adhesion on it and after rolling, return to the blood stream. (**IIa**) Upstream pro-inflammatory stimuli (cytokines, PAF, etc.) induce PAF synthesis and expression of the PAF-R on the membranes of endothelial and blood cells. (**IIb**) Binding of PAF to its receptor on the membranes of these cells further induces the PAF cycle-related amplification of the initial inflammatory stimuli, which is achieved through the expression of inflammation-related genes and the subsequent production and release of various downstream mediators, such as PAF itself and several other mediators of inflammation including eicosanoids, cytokines, growth factors, further oxidative stress (ROS, RNS, Ox-LDL, and Ox-PL), and selectins and integrins in the membranes of activated endothelial cells and leukocytes. (**III**) If unresolved, the PAF cycle-related inflammatory activation of endothelial cells leads to tight adhesion of leukocytes on the activated endothelium and subsequent migration of these leukocytes and Ox-LDL to the subendothelium. There, the crosstalk of key-junction inflammatory mediators such as PAF within the developing plaque microenvironment, with a panel of inflammatory cells of both the innate and adaptive immune system, favours inflammatory phenotypes in these cells and perpetuates a continuous inflammatory milieu, leading to the differentiation of monocytes to macrophages, which engulf Ox-LDL and further transform to foam cells; thus, facilitating the onset, increase, and expansion of atherosclerotic plaque. (**IV**) Although plaques can grow to a sufficiently large size to compromise blood flow, most of their clinical complications are attributable to arterial occlusion due to plaque erosion or rupture. Vulnerable plaques are typically large, with a necrotic core covered by a thin fibrous cap, and they contain high levels of inflammatory immune cells. Gradually accumulating foam cells die in the intima due to inflammation-induced apoptosis, and when not promptly disposed of, become necrotic, progressively leading to the formation of a thrombogenic and pro-inflammatory necrotic core with cholesterol crystals. In addition, the thin layer of the fibrous cap easily ruptures due to PAF-related inflammatory and atherothrombotic stimuli. Thus, as the plaque continues to develop, it can become unstable and rupture, leading to major cardiovascular event. PAF: platelet-activating factor; PAF-R: G-protein coupled PAF-receptor; ROS: reactive oxygen species; RNS: reactive nitrogen species; Ox-LDL: oxidised LDL; Ox-PL: oxidised phospholipids; IL-6: interleukin-6; IL-1: interleukin-1; TNFα: tumor necrosis factor-α; VEGF: vascular endothelial growth factor.

Several autacoid molecules of the microenvironment, such as PAF and its receptor, play a significant role in the pro-inflammatory activation of macrophages by oxidative stress and in the uptake of Ox-LDL by macrophages [125], since Ox-LDL contains inflammatory PAF-like oxidised phospholipids that mimic PAF and interact with these cells [105]. In addition, autacoids such as PAF and PAF-like molecules in Ox-LDL also play a significant role in the cytoskeletal reorganisation of these cells during differentiations [127], as macrophages engulf and retain large molecules such as Ox-LDL, oxidised phospholipids, and blood cells, which have also migrated into the intima and sub-intima. The macrophages become lipid-loaded foam cells through phagocytosis, scavenger-receptor mediated uptake, and pinocytosis; the macrophages become lipid-loaded foam cells [58]. The term ‘foam cells’ both reflects the microscopic appearance of these lipid-laden macrophages and denotes early fatty streak lesions [122]. This process is outlined in Figure 4.

The interplay of PAF with other APC such as DC is also implicated in several stages of atherosclerosis. Under atherosclerotic conditions, the role of DC is to take up atherosclerosis-specific antigens, which become locally activated, and migrate out of the plaque towards either local draining or distant lymph nodes, where they induce protective anti-inflammatory T cell activation and proliferation. However, apart from their role in directing different T and B cell subsets, not all their functions have been fully elucidated or understood. Nevertheless, impaired migration of DC to lymph nodes results from inhibitory signals generated by PAF or Ox-LDL that act as a PAF mimetic, thus suppressing immunologic priming. In contrast, normal DC migration and priming can be restored by HDL or HDL-associated PAF acetylhydrolase (PAF-AH), which mediates inactivation of PAF and oxidised LDL. In this context, HDL and PAF-AH maintain a normally functional DC compartment [128]. In addition, DC produce PAF that engage the PAF-R in DC membranes during maturation, and thus the capacity of DC to present antigens to lymphocytes is downregulated, due to the induction of IL-10 and the sustained and increased PGE_2_ synthesis mediated by the PAF-R. In contrast, PAF-R antagonists, by disrupting this suppressor pathway, increase DC function and could therefore be useful in increasing efficiency of vaccines and/or treatment [129]. The above PAF effects on DC perpetuate local inflammation, decrease the activation of anti-inflammatory T-lymphocytes, and thus further increase plaque growth.

Lymphocytes, particularly T-lymphocytes, are also recruited to the vessel wall by mechanisms such as monocyte recruitment; thus, they are present in atherosclerotic lesions in parallel with macrophages, but in lower amounts. CD4+ T cells (also called Th1 cells) express pro-atherogenic roles, whereas prominent Th2 (CD8+ T cells) and Treg responses seem to exhibit unclear and still controversial anti-inflammatory effects, resulting in a reduction of atherosclerosis and/or a more favourable plaque morphology in atherogenesis. PAF and other platelet-related inflammatory mediators, such as thromboxane A_2_, serotonin, and histamine, also display Th1 cell-regulatory effects towards the Th1 response that promotes the progression of atherosclerosis and diverse effects on Th2 response [130]. Activated platelets produce a significant amount of TxA_2_, which inhibits Th1 proliferation and cytokine production [131], while they also express PAF-R, and PAF can enhance Th1 cytokine production [130,132].

PAF can also promote differentiation of Th17 cells that are present in atherosclerotic lesions, which can induce cytokine production by these cells. Activated platelets and platelet thrombi create a unique microenvironment with counteracting mediators for Th17 polarisation by secreting substantial amount of PAF, TGFβ, and IL-1β [130]. However, the role of Th17 also remains controversial, as both atherogenic, as well as atheroprotective, effects have been reported [59]. Nevertheless, both PAF and Ox-LDL that mimic PAF and the PAF-R have the capacity to induce atherogenesis due to activation of T-cells and monocytes/macrophages [133]. These events lead to an expansion of atherosclerotic plaque burden and perpetuation of the pathogenic T-cell response.

Overall, there is intricate interplay and crosstalk between a panel of inflammatory cells of both the innate and adaptive immune system. When key-junction inflammatory mediators within the developing plaque microenvironment are increased, there is favour towards inflammatory phenotypes in these cells, which perpetuates a continuous inflammatory milieu, leading to further increase and expansion of the atherosclerotic plaque. Subsequently, the intimal thickness increases, and blood flow is eventually impaired. Gradually accumulating foam cells die in the intima through inflammation induced apoptosis. When these cells are not promptly disposed of they become necrotic, progressively leading to the formation of a thrombogenic and pro-inflammatory necrotic core containing cholesterol crystals [58].

##### The Overgrowth and Instability of Plaques and Subsequent Acute Cardiovascular Events

During plaque growth and expansion, SMC migrate from the media to the intima and proliferate, forming a fibrous cap from extracellular matrix deposition, where activated lymphocytes and calcium deposits are found. Although plaques can grow to a sufficiently large size to compromise blood flow, most of their clinical complications are attributable to arterial occlusion due to plaque erosion or rupture. Vulnerable plaques are typically large with a necrotic core covered by a thin fibrous cap and contain high levels of inflammatory immune cells [122]. The thin fibrous cap easily ruptures, as there are areas of the plaque where few SMC are present, and macrophages exist in abundance. This is because inflammatory cells cause the death of SMC, which are the main source of collagen that produce and maintain the fibrous cap. PAF is also implicated in the release of several proteases from leukocytes, such as elastase, that degrade the vessel’s extracellular matrix components of the intima, which may lead to plaque rupture [58]. As the plaque continues to develop it can become unstable and rupture, leading to a major cardiovascular event such as myocardial infarction, stroke, or congestive heart failure, depending on the location of the rupture.

Platelets are critical effectors in the development, progression, and resolution of the final stages of atherosclerosis, and plaque rupture, which is responsible for acute coronary disorders and stroke, not only due to their direct effects on the endothelium but also by acting as a ‘bridge’ for other cells within the vascular system [119,121]. Plaque rupture occurs under inflammatory cascades and atherothrombosis through an interplay of platelet-leukocyte aggregates. Upon vessel injury (i.e., plaque rupture), platelets readily adhere to damaged endothelium, and this binding event facilitates further activation and discharge of activating factors stored in platelet granules. Such platelet secretory components include membrane ligands and several chemokines such as PAF that play a role in further recruitment of leukocytes, additional platelets, or other blood cells to the vessel wall [121]. Platelet adhesion under conditions of high shear stress, which occurs in stenotic atherosclerotic arteries, is central to the development of arterial thrombosis. Therefore, precise control of platelet adhesion must occur to maintain blood fluidity and to prevent thrombotic complications [119].

##### Concluding Remarks on PAF in Atherosclerosis and CVD

The potent pro-inflammatory mediator, PAF, and its related PAF/PAF-R pathways are key-junctions of the inflammatory milieu during all stages of atherosclerosis and subsequent CVD. Some biochemical mechanisms involved include the pro-inflammatory induction of endothelial dysfunction, oxidative and nitrosative stress, increased platelet reactivity, recruitment/tight-adhesion, and trans-endothelial cell migration of inflammatory cells from the circulation, differentiation of pro-inflammatory monocytes to inflammatory macrophages, induction of macrophage uptake of Ox-LDL, foam cell formation, induction of plaque growth, plaque instability that leads to eventual plaque rupture, and subsequent cardiovascular events. Outcomes from multiple animal model experiments and several clinical studies have also outlined the crucial role of PAF in atherosclerosis due to its elevated levels and its inflammatory interplay and crosstalk with several cells in the pathogenesis of cardiovascular disorders. Clinical studies that have evaluated the role of PAF as a predictor of CVD have also been reviewed [89].

PAF-R antagonists have been tested with promising results [134,135,136,137,138,139,140], however the most prominent beneficial outcomes against atherosclerosis development and CVD were found when food-derived PAF inhibitors such as those present in the foods of the Med-diet. These molecules beneficially inhibit PAF activities and modulate its metabolism towards homeostatic PAF levels [103,140,141,142,143,144,145]. Many of these components are present in olive oil, wine, fish, and dairy products (Table 1). Interestingly, the administration of polar lipid extracts from fish or olive oil to hypercholesterolemic rabbits lead to the regression of atherosclerotic plaques [103,142,143,144,145]. These results clearly outline that targeting inflammation and its key-junctions such as the PAF/PAF-R pathways and PAF metabolism provide beneficial outcomes against atherosclerosis and CVD, even without targeting hypercholesterolaemia. Thus, by targeting inflammation, the cause of these disorders through non-toxic approaches such as the Med-diet and by not targeting single risk factors (such as hypercholesterolaemia) seems to provide preventive and protective beneficial results against atherosclerosis and CVD.

#### 3.2.2. The Role of PAF in Cancer and Metastatic Angiogenesis

Cancer is the second leading cause of death in developed countries. New blood vessel formation penetrating solid tumours seems to be required for their growth and metastasis. Production of PAF and overexpression of PAF-R are implicated in the tumour-endothelium interplay during cancer growth, invasion, and metastasis in several types of cancer [57,114,169,170,171]. PAF and PAF-R are also involved in tumour growth that is associated with immunosuppression [172,173,174], while the crosstalk between PAF/PAF-R pathways and growth factors receptors pathways suggests a potentially important signalling link between inflammatory and growth factor signalling in cancer [173,174,175].

It is not yet fully understood whether the initial levels of PAF in the tumour microenvironment originate from migrated inflammatory circulating cells as a response, or by activated endothelial cells in the vessels neighbouring tumours, or by the tumour cells themselves. However, there is correlation between the malignancy of cancer cells and PAF production and PAF-R expression. It seems that the production and accumulation of PAF in the tumour microenvironment originates from the coexistence of two or of all these procedures and/or by the inactivation of PAF-AH. For example, the PAF basic biosynthetic enzymes such as LPCAT1 (an isoform of lyso-PAF-AT) are overexpressed in several cancer cells and correlated with cellular invasiveness and migration. Therefore, LPCAT1 seems to contribute to tumour growth and metastasis in these types of cancer [176,177]. Moreover, endothelial cell PAF production results in enhanced inflammatory cell recruitment, while endothelial accumulation of PAF by PAF production and inactivation of PAF-AH plays also a role in cancer cell migration to distal locations [178]. In addition cigarette smoking, a classic risk factor for several cancers, contributes to metastatic disease via production of PAF and PAF-like molecules in lung tumours [174], while smoking related inhibition of breast cancer cell PAF-AH results in PAF accumulation and a subsequent increase in cell motility, tumour growth, and metastasis [178,179].

Independently of the origin, the presence of PAF in the microenvironment of tumours activates cancer cells and endothelial cells to further amplify the production of both PAF, angiogenic factors, and increased expression of their receptors on cell-membranes, including the PAF-receptor, leading to a PAF cycle and further induction of several PAF/PAF-R related cascades. These cascades, in coordination with angiogenic cytokines and growth factors, enhance the initial signal and induce morphological alterations and cellular activities such as growth proliferation and motility, expression of adhesion molecules, extracellular matrix breakdown, migration, and endothelium reorder that leads to the formation of distinct neoplastic vessels in the tumour microenvironment [57]. All of the above result in the onset and development of tumour-induced angiogenesis and metastasis [57]. For example, in pancreatic cancer, PAF overexpression leads to cell proliferation and tumourigenesis through the PAF/PAF-R related MAPK signalling pathway, causing neoplasia [170]. In addition, PAF and PAF-like molecules are in part transported by tumour-derived extracellular vesicles, which play an important role in intercellular communication through PAF-R expressed by a variety of microenvironmental cells and endothelial cells, favouring metastasis [172]. Apart from its crucial role in cancer metastasis, which has been extensively reviewed [57], recent outcomes have demonstrated that PAF is also implicated in immunosuppression-related cancer induced by UV-irradiation, in which UV-induced production of PAF and PAF-like molecules and the expression of PAF-R activates systemic immune suppression and delays DNA repair [93].

On the other hand, PAF and its receptor have been beneficially associated with cell survival during radiotherapy or chemotherapy, by proliferative signals on the surviving cells that are induced by apoptotic cells. These signals take place through mechanisms dependent on the activation of PAF-R related pathways of NF-kB, such as up-regulation of anti-apoptotic factors and decrease of the cytotoxic effect of chemotherapeutic agents, thereby contributing to cell survival [172]. However, recent studies have demonstrated that during cancer therapies (i.e., irradiation of carcinoma cells or chemotherapy), PAF-R ligands can be generated that further aggravate immune suppression and, when bound on the PAF-R of cancer cells, induce anti-apoptotic factors that protect the tumor cells from death induced by these treatments, while the combination of radiotherapy with PAF-R antagonists could be a promising strategy for cancer treatment [173,180].

In several cancer types, PAF through the NF-kB pathway controls the expression of genes that take part in processes that lead to metastatic angiogenesis on one hand, while on the other hand it results in apoptosis of cancer cells, during the immune response and haematopoiesis during chemotherapies and radiotherapies [57]. It seems that PAF is a unique growth regulator with apparently diverse functions; PAF, like NF-kB, seems to promote distinct biological processes, and these dual actions of PAF may relate to the point of action in the cell cycle [57]. The timing, space, and quantity of its production play a significant role in the malignant or beneficial direction of its effects. Understanding how conditions and factors that control timing, location of activity, and the quantity of PAF levels and how these relate to the metabolic enzymes of PAF is of great importance.

PAF-R antagonists have exhibited promising results in vitro and in vivo as anti-angiogenic molecules in several cancer cells and tumours, but also by reducing persistent pain during cancers [57,114,181,182]. In addition, the combination of chemotherapy and classic PAF-R antagonists seems to reduce the tumour volume and cause higher tumour regression when compared to each treatment alone [172,180]. Recently, synthetic glycosylated alkyl-phospholipids that act as PAF agonists and antagonists have exhibited promising antiproliferative outcomes and are now regarded as and can be new class of anti-tumour drugs [183]. However, apart from using synthetic or classic PAF antagonists, a dietary profile rich in bioactive molecules and food-derived PAF inhibitors such as those present in foods of the Mediterranean diet seems to provide beneficial preventive and protective effects against development, growth, and metastatic manifestations of cancer cells by inhibiting PAF activities and/or modulating its metabolism towards homeostatic PAF levels [57,137] (Table 1).

#### 3.2.3. The Role of PAF in Glomerulosclerosis and Renal Disorders

PAF has been characterised as one of the main inflammatory mediators implicated in renal pathophysiology [184]. Production of PAF in the kidney can potentially be attributed to infiltrating inflammatory cells, but mostly to resident renal cells such as the mesangial cells of glomeruli [81,185]. Once synthesised, PAF does not accumulate in renal cells, but it is secreted and thus affects mesangial cells, neighbouring podocytes, and other infiltrating cells by binding to its receptor and inducing PAF/PAF-R pathways. In the kidney, PAF-R mRNA is ubiquitously expressed, and a gradient of its expression levels seems to exist; it is higher in the renal cortex, lesser in the outer medulla, and much lesser in the inner medulla, while within the nephron, the glomerulus demonstrates the highest PAF-R expression [78]. PAF infusion affects renal hemodynamics and glomerular permeability, resulting in changes in filtration rate and proteinuria [78].

Apart from the physiological effects of PAF, its increased levels and overexpression of PAF-R in kidney are involved in the pathogenesis and progression of renal damage and acute renal failure [78,184,186,187]. Thus, PAF is implicated in antibody- and complement-mediated glomerular injury, in antithymocyte antibody-induced glomerular damage and other experimental models of immune renal damage, and in patients with lupus nephritis and IgA nephropathy [78]. PAF participates in the development of kidney graft dysfunction, namely, transplant rejection chronic transplant nephropathy and immunosuppressive drug-mediated nephrotoxicity [78]. PAF is also implicated in drug-related renal damage of different causes, such as cyclosporin A, glycerol, gentamicin, and cisplatin [78].

However, the most important role of PAF in renal dysfunction is its implication in the onset and progression of glomerulosclerosis, a renal disorder that shares common features with atherosclerosis and can lead to organ failure. Crosstalk between several renal cells of the glomeruli, such as the mesangial cells and podocytes, takes place during this disorder and the PAF/PAF-R pathways form key junctions during all steps. It has been proposed that PAF might be one of the chemokines released by mesangial cells that mediate their communication with podocytes. PAF enhances its own receptor expression [188], through which it stimulates multiple downstream inflammatory signalling pathways, mostly in mesangial cells, leading to the release of AA metabolites and subsequent prostanoid and thromboxane generation, leukocyte recruitment, mesangial cell contraction, intracellular lipid accumulation, and transforming growth factor (TGF)-β mediated upregulation of extracellular matrix production. All of these molecular events potentially culminate in the development of glomerulosclerosis and fibrosis, which are key feature of progressive renal disease, regardless of the primary cause [78,188,189]. In addition, PAF promotes inflammatory infiltration of the glomerulus, since it functions as a chemoattractant, and it increases adhesion of polymorphonuclear leukocytes and monocytes to mesangial cells through integrins [78]. PAF increases the expression of the LDL-receptor and scavenger receptors in mesangial cells, and thus causes an increased uptake of lipids and their accumulation in mesangial cells, leading to the formation of foam cells, which is an important stage of glomerulosclerosis and a key factor that participates in the initiation and progression of lipid-mediated renal injury [78,188].

Several PAF-R antagonists have been used in several of the aforementioned renal disorders with promising results [78,137]. However, apart from using classic PAF antagonists, recent results have highlighted the protective role of a dietary profile rich in bioactive molecules, antioxidants, and food-derived PAF inhibitors such as those present in the Mediterranean diet through beneficially inhibiting PAF activities and/or modulating its metabolism towards homeostatic PAF levels [80,81] (Table 1). In addition, the use of vitamin D or vitamin-D analogues as treatment in haemodialysis patients has also exhibited similar beneficial effects, since such a treatment strongly inhibits PAF and thrombin activities, affects PAF metabolism towards equilibrating PAF levels, and reduces circulating levels of IL-8, IL-1β, and TNF-α [79]. As reducing dietary cholesterol levels may be ineffective, such outcomes have further supported the notion of using full-fat products such as dairy products and non-low-fat products, since the full-fat dairy products exhibit higher bioavailability of high-value nutrients such as bioactive polar lipids and vitamin D, which both possess strong anti-inflammatory and protective properties [3].

#### 3.2.4. The Role of PAF in Cerebrovascular and Central Nervous System Disorders

PAF and the PAF/PAF-R pathways are also present in the CNS, where they exhibit a number of diverse physiological and pathological functions. PAF is synthesised in neuronal cells throughout the CNS, while these cells also express the PAF-R [190,191]. When present at normal concentrations, PAF is a modulator of many CNS processes, ranging from long-term potentiation to neuronal differentiation [113,191]. Excessive levels of PAF appear to play an important role in neuronal cell injury and in various inflammation-related CNS pathological conditions, such as neuroinflammatory cascades implicated in depression and neurodegeneration, Alzheimer’s disease, stroke, ischemia-reperfusion injury, spinal cord injury, multiple sclerosis, Parkinson’s disease, neuropathic pain, epilepsy, central malaria, meningitis, depression, cognitive deficits, and HIV-induced neurotoxicity [190,191,192]. Increased PAF synthesis through the PAF/PAF-R pathways can cause a severe inflammatory response, reduction of biological membrane integrity, ROS and RNS formation, expression and release of cytokines, alterations in blood–brain barrier permeability and the permeability of blood vessel walls, activation and recruitment of inflammatory and immune cells, secretion of cell-specific proteins, induction of cell apoptosis through specific signalling pathways, and other pathological responses [113,190,191,192,193]. PAF accumulation in CNS diseases exacerbates the inflammatory response and pathological consequences, while application of PAF inhibitors or PAF-R antagonists significantly reduces inflammation, protects cells, and improves the recovery of neural functions by blocking the PAF pathway [191,192,194]. Several PAF inhibitors of natural origin have also exhibited beneficial outcomes in CNS disorders, especially ginkgolides that are derived from *Ginkgo biloba* [137,195]. However, further studies are required to establish the mechanisms surrounding how a healthy diet can improve systemic inflammation associated with the PAF pathway and CNS disorders.

#### 3.2.5. The Role of PAF in Allergies and Asthma

Anaphylaxis is defined as a severe, life-threatening, systemic or general, immediate reaction of hypersensitivity, with repeatable symptoms caused by a dose of stimulus that is well tolerated by healthy persons [196,197]. Recently, PAF and PAF-AH have been reported as clinically valuable biomarkers of anaphylaxis [196], since PAF produced and released by mast cells, basophils, neutrophils, eosinophils, fibroblasts, platelets, endothelial cells, and even cardiac muscle cells plays an important role in anaphylaxis and several other allergic reactions, from allergic rhinitis to asthmatic complications [67,196,197,198,199,200,201,202]. Eosinophils, mast cells, and basophils are implicated in allergies, and they have the capacity to influence each other’s functions through a crosstalk, where other mediators such as PAF are also implicated [198,199,200,203]. PAF increases the production of eicosanoids, ROS, cytokines, growth factors, platelet-derived growth factor (PDGF), RANTES, and degranulation of eosinophils, while it also acts as a chemoattractant for these cells, and, via integrins, it increases their adhesion to vascular endothelium. Mast cells not only produce PAF, but they can also be activated by it through the PAF/PAF-R pathways. Thus, exposure of mast cells to PAF leads to the induction of specific functions in these cells such as degranulation of their granules via neuropeptides and PAF-dependent release of histamine. In fact, the greater the levels of PAF in mast cells microenvironment, the more enhanced the release of histamine. At the same time, PAF-activated myocardial mast cells locally release factors responsible for cardiac dysfunction and hypotension that occur in severe anaphylactic reactions [197,200].

Increased levels of PAF correlate with the severity of allergic systemic reactions. Thus, PAF has been found to be involved in several allergic and anaphylactic reactions and shock, in inflammation of bronchi and bronchial asthma and in asthmatic patients’ bronchoconstriction, in mucus hypersecretion, in allergic rhinitis, and in urticaria pathogenesis [200]. Several studies have shown that PAF can enhance obstructive changes of bronchi by stimulation of allergic inflammation of the respiratory tract epithelium, while PAF can also increase the permeability of skin’s capillaries and induces the development of wheals, flare, and inflammatory reactions in the skin through its interactions and crosstalk of the aforementioned inflammatory cells involved in these pathological conditions [200].

The protective role of PAF-AH in reducing PAF levels is usually highly diminished through allergic reactions [196,200], while administration of recombinant PAF-AH in animal models exhibited protective results and reduced mortality due to anaphylactic reactions [196], implying that modulation of PAF metabolism towards homeostatic PAF levels can also provide beneficial outcomes in these disorders too. In addition, specific PAF-R inhibitors have been used in several allergy-related disorders [137], and even specific anti-allergic drugs were designed and are currently used according to their anti-PAF effects [204,205], while combination of PAF inhibitors with other therapies such as antihistamines provided better outcomes [137,198,199,201]. However, further studies are required to establish the potential of a healthy diet to improve systemic inflammation associated with the PAF pathway and allergic complications.

#### 3.2.6. The Role of PAF in Chronic Infections and Inflammation-Associated Comorbidities

Inflammatory and immune responses are central to protecting against most infectious agents. However, the pathogenesis and tissue damage after infection are not usually related to the direct action microorganisms and of their replication, but instead to altered immune and inflammatory responses triggered following contact with the pathogen. Many diseases develop as an adverse consequence of an imbalanced inflammatory response; thus, chronic and unresolved infections are usually accompanied by chronic and unresolved inflammatory manifestations and comorbidities [206]. PAF and PAF-like molecules are implicated in inflammatory manifestations occurring in several infections [206,207], such as HIV [69,70,71,72,73,74,85], leishmaniosis [208], periodontitis [75,76,77], or even in sepsis [67,209]. The relationship between increased PAF levels, overexpression of PAF-R, and the PAF/PAF-R pathways with several other mediators such as cytokines and inflammatory cells leads to the progression of such diseases and their related comorbidities.

The most common coexistent diseases associated with chronic infections are CVD, CNS disorders, and tumour malignancies, which are usually promoted by increased levels of PAF and PAF-related continuous and unresolved inflammation [57,67,68,69,70,71,72,73,74,75,76,77,85,208]. In addition, PAF seems to act in synergy with infectious agents to initiate and propagate the disease process, i.e., viral load in HIV-infected patients was positively correlated with PAF synthesis and levels, while viral products such as Tat-protein induce PAF synthesis and PAF-related HIV-induced non-AIDS comorbidities, such as CVD, Kaposi sarcoma, neurodegeneration, and dementia [69,70,71,72,73,74].

Several PAF inhibitors have been used in infectious diseases with promising results, mostly in relation to their deterioration of the PAF-related chronic inflammatory manifestations [67,71,74,85,137,207,209,210,211]. However, in the case of severe sepsis, clinical trials using recombinant human PAF-AH or PAF-R antagonists failed to reduce the mortality of severe septic patients, although a substantial reduction in organ dysfunction was achieved [206]. Drugs administrated in such infectious pathologies have also been thoroughly screened for potential dual actions against both the infectious agent and PAF activities and synthesis. Several antiretrovirals and their combinations in highly active antiretroviral therapy have been found to exhibit beneficial outcomes in HIV infected patients through their capabilities to inhibit PAF activities and to influence PAF metabolism towards reduction of PAF levels in vitro and in vivo, while similar outcomes have also been found for several antibiotics [68,69,71,73]. Nevertheless, inhibition of PAF activities and modulation of PAF metabolism towards homeostatic PAF levels seem to be useful therapeutic targets with which to interfere with inflammatory damage that follows an infection, and thus they may reduce the risk of several comorbidities in infectious disorders. Although there are several studies published on the importance of a healthy diet for infection prevention, further studies are required to establish the potential role of healthy eating to improve systemic inflammation associated with the PAF pathway and related complications during chronic infections.

#### 3.2.7. The Role of PAF in Various Inflammation-Related Chronic Diseases

PAF has also played a role in several other inflammation-related chronic diseases and their related comorbidities, including types I and type II diabetes mellitus [212,213,214], acute pancreatitis [215,216], liver injury [217], inflammation-related intestine tissue dysfunction such as necrotising enterocolitis [218,219], inflammatory ocular diseases [220], vascular dysfunction during acute lung injury [221], and autoimmune disorders, such as rheumatoid arthritis, systemic lupus erythematosus, multiple sclerosis, inflammatory bowel disease, and Crohn’s disease [222,223,224].

Several PAF inhibitors have been used in these inflammation-related diseases with promising results [137,216,217,222,225,226]. These effects were mostly due to the deterioration of PAF-related chronic inflammatory manifestations present in these disorders. However, apart from using synthetic or classic PAF antagonists, a dietary profile rich in bioactive molecules, antioxidants, and food-derived PAF inhibitors such as those present in foods of the Mediterranean diet may provide beneficial preventive and protective effects against these diseases too, through beneficially inhibiting PAF activities and/or modulating its metabolism towards homeostatic PAF levels. For example, the consumption of components of the Med-diet or a traditional Greek Mediterranean diet can reduce PAF-related inflammatory outcomes such as platelet activity in patients suffering from type II diabetes mellitus and metabolic syndrome, but also in healthy subjects. This has been attributed to the presence of PAF inhibitors among other possible effects, and these effects can occur over a short period of time [227,228,229]. In addition, the use of probiotics has exhibited beneficial effects against necrotising enterocolitis [218]; this is unsurprising, as fermented dairy products, which are also components of the Med-diet, are rich in PAF inhibitors and have also exhibited beneficial outcomes in several inflammation-related intestine dysfunctions [3].

### 3.3. Targeting the PAF Pathways and Metabolism – Beneficial Outcomes of the Mediterranean Diet

Common junctions in the mechanistic crosstalk of inflammatory mediators, signalling pathways, and cellular interactions that occur during chronic and unresolved inflammatory manifestations seem to be promising therapeutic targets for the prevention and treatment of inflammation-related chronic diseases. Drug-based therapeutic interventions targeting inflammatory mediators such as cytokines (i.e., by using specific antibodies against pro-inflammatory cytokines and their receptors) and eicosanoids (i.e., by using specific inhibitors of COX-1 and COX-2) have also been proposed, and relative trials such as CANTOS are still in progress. However, such approaches can sometimes provide undesirable effects and may leave the individual immunocompromised and at a greater risk of infections, since disruption of the physiological balance seems to be a risky strategy [230,231], which is clearly behind the multifaceted effects of such mediators.

On the other hand, since PAF and its related inflammatory cascades belong to the most vital joint mechanistic pathways of inflammation-related chronic disorders, the exploration of possible therapeutic approaches targeting PAF and its related pathways may provide better outcomes. Focus initially was given to the PAF/PAF-R interaction, thus inhibiting the exacerbation of the complex PAF inflammatory pathways [89,134,135,136,137]. There are several agonists of synthetic and natural origin [57,89,134,135,136,137,140,232], which can competitively or noncompetitively displace PAF from its binding sites on PAF-R and thus directly inhibit the PAF/PAF-R related pathways and PAF activities. Furthermore, other similar molecules can indirectly affect the PAF/PAF-R pathways by affecting the up-stream and/or downstream microenvironment of PAF-R, lipid, rafts, and other related cellular receptors.

Even though such specific PAF antagonists for the PAF/PAF-R pathway have exhibited promising results, the most prominent beneficial effects have been derived from polar lipids and polar lipid extracts derived from several foods, particularly from foods in the Med-diet (Figure 1B and Table 1) [56,57,80,81,103,142,146,147,148,149,150,151,152,153,154,155,156,157,158,159,162,163,164,165,233,234]. These Med-diet polar lipids exhibit in vitro and in vivo anti-inflammatory activities through either directly or indirectly inhibiting the PAF/PAF-R pathways and thus PAF activities, but also by downregulating its levels through modulating the activities of key metabolic enzymes of PAF by either upregulation of the PAF catabolic enzymes and/or the downregulation of the basic PAF biosynthetic enzymes (Figure 2C and Figure 3C, and Table 1) [57,80,81,103,146,148].

Notably, the uptake of such dietary polar lipids seems to beneficially affect the functionality of HDL lipoproteins, especially in atherosclerotic conditions. HDL has been characterised as the ‘good’ cholesterol, since not only does it remove excess cholesterol from the blood stream and from atherosclerotic plaques, but it has also exhibited anti-inflammatory and antioxidative properties through a plethora of cardioprotective enzymes bonded in HDL, including the aforementioned PAF-AH enzyme activity, which is the main catabolic enzyme of PAF [110]. These HDL-associated activities contribute to the maintenance of endothelial cell homeostasis, which protects the cardiovascular system [235]. Plasma PAF-AH is also found in atherosclerotic lesions, since it comigrates there along with the lipoproteins (i.e., LDL), where it is incorporated. Plasma PAF-AH (Lp-PLA_2_) mainly plays an anti-inflammatory role in leukocyte/platelet/endothelium activation and seems to suppress atherogenic changes in plasma lipoproteins (such as LDL) by promoting the catabolism of PAF and by removing oxidised phospholipids present in Ox-LDL, including oxidised phospholipids that mimic PAF, which are generated by oxidative modifications of lipoproteins such as LDL during pro-atherogenic and atherosclerotic events [107,109,110]. Thus, during inflammatory cascades that cause increased PAF levels, this isoform of PAF-AH (LpPLA_2_) seems to be activated as a homeostatic mechanism to downregulate these events by downregulating the levels of PAF and oxidised phospholipids as a terminator signal [236]. However, during persistent and prolonged inflammatory cascades and persistent oxidation of plasma lipoproteins, plasma PAF-AH is progressively inactivated (plasma PAF-AH is incorporated mainly in LDL) and loses its capacity to protect against the pro-inflammatory actions of PAF and PAF-like lipids [98]. Because of that, but also because of the activities of the oxidised sub products of PAF-AH actions in LDL oxidised phospholipids, the use of plasma PAF-AH as an atherogenic biomarker and therapeutic target has been debated [109,236].

Nevertheless, HDL and its enzymes, including PAF-AH, seem to protect against these manifestations. The focus has been placed on increasing HDL levels as one of the main goals of dietary interventions and drug administration for cardioprotection [110]. Dietary intake of bioactive polar lipids, particularly those baring ω-3 PUFAs, increase HDL levels and the incorporation of such anti-inflammatory and antioxidant dietary polar lipids to HDL, thus providing an additional protective mechanism by increasing plasma PAF-AH activity and protecting the HDL enzymes (such as PAF-AH) from oxidation-related inactivation. This is in agreement with the beneficial in vitro and in vivo effects of several dietary polar lipids, especially on PAF metabolism and HDL biofunctionality [56].

PAF can generate ROS, and oxidative stress is a key feature of the atherothrombotic processes in the pathology of CVD. Therefore, it is important to recognise that foods of the Med-diet such as fruit and vegetables are high in chemical constituents, many of which are regarded as powerful antioxidants, such as vitamins A, C, and E [237]. Despite positive findings from in vitro studies, clinical trials have consistently failed to show a benefit for the use of antioxidants, as associations between plasma concentrations of antioxidant vitamins and protection against CVD have proved elusive, and large interventional trials have failed to conclusively show any benefit of their administration [238,239,240,241]. Despite this, the European prospective investigation into cancer and nutrition (EPIC) Norfolk study found that increased plasma concentrations of vitamin C were inversely associated with CVD-related mortality and all-cause mortality. The study found that this increase was due to increased intake of fruit and vegetables, which led to an approximate 20% decrease in CVD mortality [242]. However, a meta-analysis has shown that vitamin C supplementation did not reduce cardiovascular events; thus, the antioxidant effects of vitamin C were not responsible for the beneficial effects of increased consumption of fruit and vegetables [243]. A large-scale, 20-year study found that diets rich in vitamin C were associated with a lower incidence of stroke, but no coronary heart disease in the elderly [244]. Considering these findings, it may be the case that vitamin C may not be the active agent that induced the effects witnessed in the Norfolk study, but although eating fruit and vegetables will increase plasma vitamin C levels, the effects observed may be through other fruit- and vegetable-derived nutrients [241], or synergism between multiple nutrients that affect different mechanisms including inflammation through the mechanisms of the PAF pathways [245].

The bioavailability of vitamins, phenolic compounds, and other antioxidants is often cited as the main reason that in vitro and ex vivo studies do not seem to agree [237]. For instance, some antioxidants such as phenolic compounds are effectively screened out by the gut of rapidly metabolised and excreted [246]. Plasma concentrations of phenolic compounds are typically in the nanomolar range—too low to have a direct impact on antioxidant capacity [241]. However, many of these antioxidant molecules do seem to possess beneficial effects upon consumption, including the idea that they induce indirect antioxidant activity by acting as a mild toxin to stimulate a general xenobiotic and/or an antioxidant response [237]. Further research is required to elucidate the effects of certain biomolecules against ROS and inflammatory pathways. 

Overall, the protective outcomes of the adoption of Med-diet towards chronic diseases seem to be associated with the pleiotropic beneficial effects of its bioactive microconstituents that are not only limited to increasing plasma-HDL levels, functionality, and providing better stability against oxidation, but mainly on their effects on the levels, activities, and metabolism of key-inflammatory mediators such as PAF [56,57]. However, more in vivo results are needed in several chronic disorders and their inflammation-related manifestations in order to further support these findings. In particular, clinical trials implementing dietary patterns such as the Med-diet that are rich in bioactive polar lipids interacting with the PAF/PAF-R pathways and metabolism are required to gain further insight into the role of PAF in chronic diseases.

## 4. Conclusions

In this review, we clarify the roles of risk factors, such as plasma cholesterol and the importance of causative agents for chronic diseases, namely chronic and unresolved inflammation and its manifestations. Instead of cholesterol, targeting and treatment of inflammation will lead to lower side effects in chronic disorders. The overall outcomes and the extensive paradigms of the beneficial effects of the Mediterranean diet against the inflammatory milieu, without any reported side effects so far, have radically shifted attention away from the lipid-centric hypotheses and the subsequent trends for targeting cholesterol towards more effective approaches against inflammation, which is the causative factors of chronic diseases.

Therefore, the causative role of inflammation in the onset and progression of several chronic disorders is summarised with respect to the role of PAF and its related inflammatory cascades, which seem to serve as common junctions of the inflammatory milieu. The coexistence of several risk factors seems to upstream pro-inflammatory stimuli (i.e., cytokines, oxidative stress, PAF itself, etc.), leading to increased levels of PAF that, through the PAF/PAF-R pathways, attenuate the initial signal, while downstream inflammatory cascades, in combination with inactivation of homeostatic mechanisms such as PAF catabolism, can result in chronic and unresolved inflammatory manifestations and related chronic disorders.

Common junctions, such as PAF and its related inflammatory pathways, seem to be promising therapeutic targets for the prevention and treatment of the onset and progression of inflammation-related chronic diseases, particularly CVD. Implementation of healthy lifestyle choices based on appropriate dietary interventions and exercise have exhibited beneficial outcomes and health benefits, without noticeable side effects. Adoption of dietary patterns such as the Med-diet provides bioactive food microconstituents with pleiotropic beneficial effects that are not limited to decreasing co-absorption of cholesterol and increasing plasma HDL levels and functionality, but mainly by providing better stability against oxidation and inflammation. Therefore, microconstituents such as polar lipids and vitamins present in foods of the Med-diet beneficially affect the levels, activities, and metabolism of key inflammatory mediators implicated in chronic diseases, including the PAF pathway, towards reducing inflammation and acquiring homeostasis, which can lead to reduced risk of inflammation-related chronic disorders.

Nature has provided us with a wide range of dietary weapons, which, if appropriately combined in dietary patterns such as the Med-diet, can beneficially contribute to improving our quality of life, health, and life expectancy by equilibrating the inflammatory milieu to normal levels and thus preventively reducing the risk of inflammation-related chronic disorders. Let us not forget the words of Hippocrates of Kos (460-377 BC), who is universally recognised as the father of modern medicine: “*Let food be thy medicine and medicine be thy food*”.

## Figures and Tables

**Table 1:** Studies on the beneficial impact of microconstituents from foods of the Mediterranean diet, such as polar lipids and vitamins, towards inflammation-related disorders, through their effects on the PAF pathways and metabolism.

Studied Food and Components	Type of Study	Results
PL of red and white wine, musts, grape-skins, and yeast	In vitro studies in WRP and in U937 macrophages, In vivo postprandial dietary interventions studies in humans	Inhibition of PAF-induced platelet aggregation and modulation of PAF metabolism towards reduced PAF levels [141,146,147,148,149,150,151]
PL of Fish (sea bass, sea bream, salmon, etc.)	In vitro studies in WRP, and in HMC, Ex vivo studies in hPRP In vivo studies in hyperlipidaemic rabbits	Inhibition of PAF-induced platelet aggregation, modulation of PAF metabolism towards reduced PAF levels, and reduction of the thickness of atherosclerotic lesions in hypercholestrolaemic rabbits [81,103,142,152,153,154,155,156,157,158,159,160], unpublished data for salmon PL
PL of olive oil and olive pomace	In vitro studies in WRP and in HMC, In vivo study in hyperlipidaemic rabbits	Inhibition of PAF-induced platelet aggregation and modulation of PAF metabolism towards reduced PAF levels, and reduction of the thickness of atherosclerotic lesions in hypercholestrolaemic rabbits and regression of the already formed atherosclerotic lesions [80,144,145,161]
PL of seed oils (soybean, corn, sunflower, and sesame oil)	In vitro studies in WRP	Inhibition of PAF induced platelet aggregation [161]
PL of Hen egg	In vitro studies in WRP	Inhibition of PAF-induced platelet aggregation [162]
PL of dairy products (milk, yoghurt, cheese, etc.)	In vitro studies in WRP and ex vivo studies in hPRP	Inhibition of PAF-induced platelet aggregation [3,163,164,165] unpublished data for bovine, ovine, and caprine milk, and yogurt and cheese in hPRP
Lipid extracts from garlic	Ex vivo studies in hPRP	Inhibition of PAF-induced platelet aggregation and de-aggregation of aggregated platelets [166]
Vitamin D and its analogues	In vitro studies in WRP and human leukocytes, ex vivo studies in hPRP and in vivo studies in haemodialysis patients	Inhibition of PAF-induced platelet aggregation and modulation of PAF metabolism towards reduced PAF levels and reduction of the inflammatory milieu (reduced levels of several cytokines) [79]
Vitamin E	Ex vivo studies in hPRP and whole blood	Inhibition of PAF-induced platelet aggregation [167,168]
Mediterranean-based meals and diets, rich in PL with anti-PAF effects	In vivo studies in humans	Reduction of PAF-induced platelet activity in patients with diabetes-II, metabolic syndrome, and healthy subjects

Abbreviations: HMC = human mesangial cells; hPRP = human platelet-rich plasma; PAF = platelet-activating factor; PL = polar lipids; WRP = washed rabbit platelets.
